# Therapeutic effects of nicotinamide mononucleotide and Indian gooseberry in oxidative stress and inflammation-induced damage on human retinal pigment epithelial cells: A pilot study

**DOI:** 10.7555/JBR.39.20250452

**Published:** 2026-05-21

**Authors:** Deokho Lee, Jolly Shamsun Nahar, Sichan Kim, Soon Sung Lim

**Affiliations:** 1 The Korean Institute of Nutrition, Hallym University, 1 Hallymdeahak-Gil, Chuncheon, 24252 Republic of Korea; 2 Department of Food Science and Nutrition, Hallym University, 1 Hallymdeahak-Gil, Chuncheon, 24252 Republic of Korea

Dear Editor,

Age-related macular degeneration (AMD) is a leading cause of visual impairment and vision loss among older adults worldwide. It is characterized by the accumulation of drusen and dysfunction or damage to the retinal pigment epithelium (RPE)^[1]^. Traditionally, AMD is classified into two clinical forms: dry and wet. Dry AMD is more common and progresses slowly, whereas wet AMD is less common but may lead to rapid vision loss due to choroidal neovascularization followed by chronic inflammation. Oxidative stress is a key contributor to both dry and wet AMD pathogenesis, as excessive reactive oxygen species (ROS) gradually damage ocular cells, ultimately triggering severe ocular inflammation. Given that dry AMD may progress to wet AMD at any stage, early intervention targeting oxidative stress and inflammation is critically important.

Although anti-vascular endothelial growth factor therapy has been introduced to improve some of the severe pathological outcomes (particularly choroidal neovascularization) in wet AMD cases, no effective treatment currently exists for other pathological processes such as oxidative stress and/or inflammation^[2]^. Therefore, novel therapeutic strategies are urgently needed to address this issue.

Nicotinamide mononucleotide (NMN) is a key intermediate in the biosynthesis of nicotinamide adenine dinucleotide (NAD^+^) in the human body. NAD^+^ plays a central role in regulating a wide range of cellular processes, including proliferation, DNA repair, and cell survival and death^[3]^. NMN supplementation has been reported to suppress age-related or metabolism-mediated deterioration across various tissues and cell types by restoring the NAD^+^/sirtuins axis, which could help alleviate oxidative stress and/or inflammatory responses^[4–8]^. Recently, supplementation with NMN has also been suggested to protect against ocular damage in several murine models of retinopathy^[9]^. However, the therapeutic effects of NMN on RPE cells still require further investigation.

Indian gooseberry (amla) is a widely used botanical with applications in the medicinal, culinary, and cosmetic industries across Asian countries. Accumulating evidence has shown that amla possesses anti-inflammatory and antioxidant properties, which can be used to treat various metabolic disorders and diseases^[10]^. Despite growing interest in its therapeutic potential, the effects of amla on ocular health remain underexplored.

In the current study, we aimed to investigate the therapeutic effects of NMN and amla against oxidative stress- and inflammation-induced damage in human RPE cells. ARPE-19 cells were cultured in DMEM/F-12 medium supplemented with 10% fetal bovine serum and 1% penicillin/streptomycin at 37 ℃ in a humidified 5% CO_2_ incubator. To induce cellular stress, ARPE-19 cells were treated with 500 µmol/L tert-butyl hydroperoxide (TBHP; Cat. #A13926.AE, Thermo Fisher Scientific, Waltham, MA, USA) and 5 µg/mL lipopolysaccharide (LPS; Cat. #tlrl-pglps, Invitrogen, Carlsbad, CA, USA). TBHP is a well-established inducer of oxidative stress, whereas LPS is commonly used to elicit an inflammatory response. NMN (Cat. #N3501, Sigma-Aldrich, MO, USA) and other drug candidates, including amla extract (Nbiotech, Hwasong, Korea), EX-527 (Cat. #E7034, Sigma-Aldrich), (+)-sodium L-ascorbate (Cat. #A4034, Sigma-Aldrich), and quercetin (Cat. #Q4951, Sigma-Aldrich), were co-treated with TBHP and LPS, depending on the experimental conditions. The viability of ARPE-19 cells was assessed using thiazolyl blue tetrazolium bromide (MTT; Cat. #M2128, Sigma-Aldrich)^[11]^. Cellular NAD^+^ levels were measured using the NAD/NADH Assay Kit (Cat. #ab65348, Abcam, Cambridge, MA, USA)^[12–13]^. The human interleukin-6 (IL-6) enzyme-linked immunosorbent assay (ELISA) (Cat. #EH2IL6, Invitrogen) was performed according to the manufacturer's instructions. The antioxidant properties of amla extract were evaluated using the DPPH (Cat. #D9132, Sigma-Aldrich) and ABTS assays (Cat. #10102946001, Roche Diagnostics GmbH, Mannheim, Germany), as detailed in our previous studies^[14–16]^. Trolox (Cat. #238813, Sigma-Aldrich) was included as a standard positive control in these assays.

Quantitative analysis of (+)-sodium L-ascorbate and amla extract was performed using an Agilent 1100 Series high-performance liquid chromatography system (Agilent Technologies, Santa Clara, CA, USA) equipped with a diode array detector. Detection was carried out at a wavelength of 244 nm. Chromatographic separation was achieved on a Zorbax SB-Aq analytical column using a mobile phase composed of formic acid in distilled water and methanol, under a standard gradient elution program. Data acquisition and processing were conducted using the ChemStation Edition software package. For quantification, a calibration curve was generated from a series of (+)-sodium L-ascorbate standards prepared by serial dilution. The concentration of (+)-sodium L-ascorbate in the amla extract was determined by substituting the sample's peak area into the resulting regression equation. Data are presented as the mean ± standard deviation. Statistical analyses were performed using one-way ANOVA followed by Bonferroni's post-hoc multiple comparison test or a two-tailed Student's *t*-test, depending on the experimental design. A *P*-value < 0.05 was considered statistically significant.

Based on our results, stress induced by TBHP and LPS significantly reduced cell viability in ARPE-19 cells (***Fig. 1A***). NMN (at concentrations ranging from 2 mmol/L to 4 mmol/L) protected ARPE-19 cells against this pathological condition. Sirtuin 1 has been proposed as a key mediator of the protective effects of NMN in various cell types^[9]^. Therefore, we reproduced this finding in our RPE cell line using 10 µmol/L EX-527 (a selective inhibitor of sirtuin 1). This concentration has been commonly used in RPE cells without unexpected cytotoxicity based on previous literature^[17–19]^. Notably, EX-527 treatment alone, without NMN, did not significantly alter cell viability. Next, we found that our stress model reduced NAD^+^ levels in ARPE-19 cells, while this reduction was suppressed by NMN treatment (***Fig. 1B***). Furthermore, EX-527 treatment did not significantly affect NAD^+^ levels, with or without NMN treatment.

**Figure 1:**
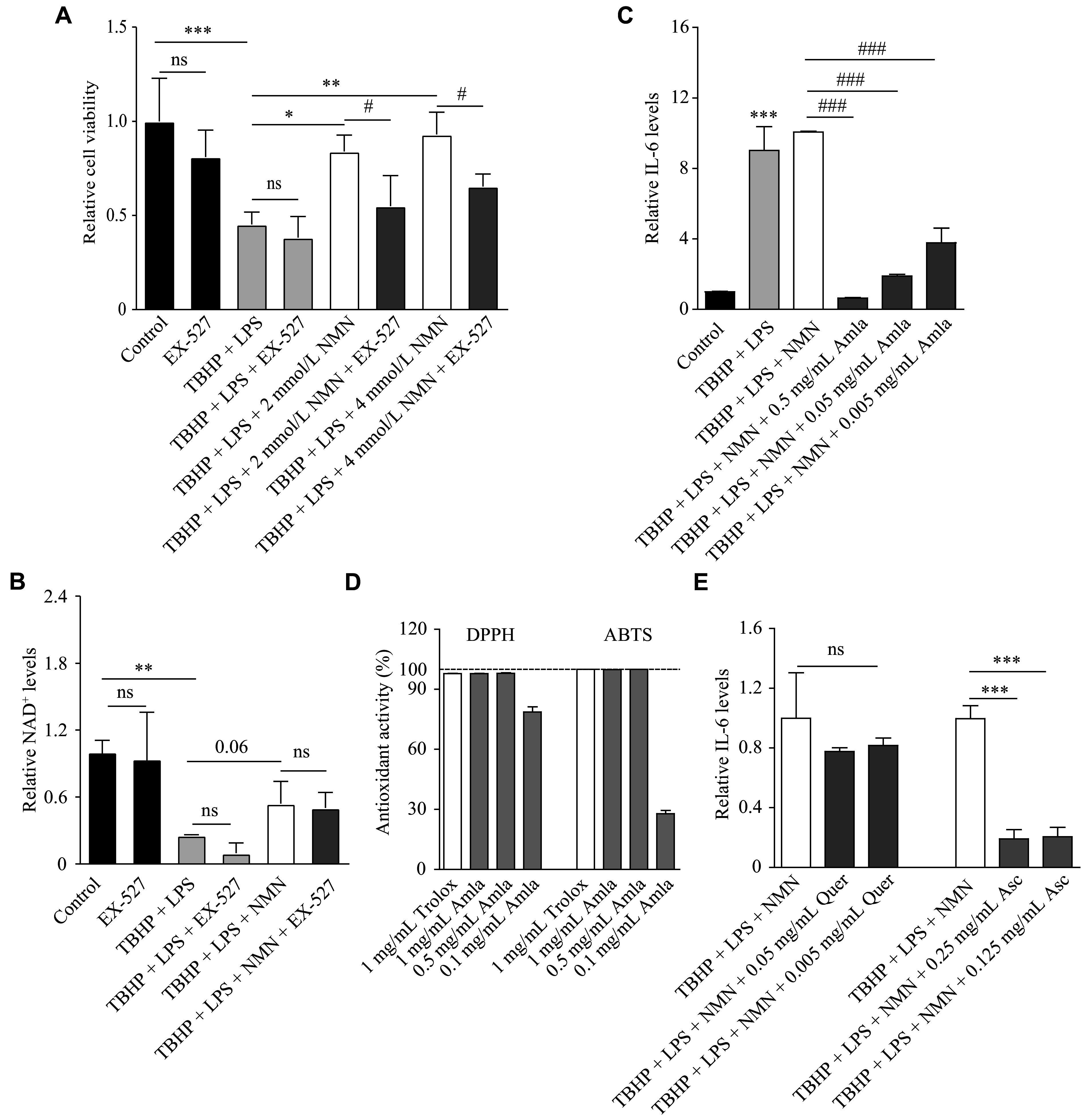
Therapeutic effects of nicotinamide mononucleotide (NMN) and Indian gooseberry (amla) on oxidative stress and inflammation-induced injury in ARPE-19 cells. A: MTT assay results of ARPE-19 cells treated with tert-butyl hydroperoxide (TBHP; 500 µmol/L) + lipopolysaccharide (LPS; 5 µg/mL) for 6 h, alone or in combination with NMN (2 and 4 mmol/L) and/or EX-527 (10 µmol/L) (*n* = 3–5 per group). ^ns^*P* ≥ 0.05, ^*^P < 0.05, ^**^P < 0.01, and ^***^P < 0.001 by one-way ANOVA followed by Bonferroni's post-hoc multiple comparison test. ^#^*P* < 0.05 by a two-tailed Student's *t*-test. B: Nicotinamide adenine dinucleotide (NAD^+^) levels in ARPE-19 cells treated with TBHP (500 µmol/L) + LPS (5 µg/mL) for 6 h, alone or in combination with NMN (4 mmol/L) and/or EX-527 (10 µmol/L) (*n* = 3–4 per group). *P* = 0.06 by a two-tailed Student's *t*-test. ^ns^*P* ≥ 0.05 and ^**^P < 0.01 by one-way ANOVA followed by Bonferroni's post-hoc multiple comparison test. C: Interleukin-6 (IL-6) levels measured by enzyme-linked immunosorbent assay (ELISA) in ARPE-19 cells treated with TBHP (500 µmol/L) + LPS (5 µg/mL) for 24 h, alone or in combination with NMN (4 mmol/L) and/or amla extract (0.005, 0.05, and 0.5 mg/mL) (*n* = 4–6 per group). ^***^P < 0.001 and ^###^*P* < 0.001 by one-way ANOVA followed by Bonferroni's post-hoc multiple comparison test. D: Antioxidant activity measured by DPPH (left) and ABTS (right) assays (*n* = 3 per group). The antioxidant effect of amla (0.1 mg/mL to 1 mg/mL) was compared with that of trolox (positive control; 1 mg/mL). E: IL-6 levels measured by ELISA in ARPE-19 cells treated with TBHP (500 µmol/L) + LPS (5 µg/mL) + NMN (4 mmol/L) for 24 h, alone or in combination with quercetin (Quer) (0.005 mg/mL and 0.05 mg/mL) or (+)-sodium L-ascorbate (Asc) (0.25 and 0.125 mg/mL) (*n* = 3–5 per group). ^ns^*P* ≥ 0.05 and ^***^P < 0.001 by one-way ANOVA followed by Bonferroni's post-hoc multiple comparison test. Data are presented as mean ± standard deviation (A–E).

As a pro-inflammatory cytokine, IL-6 has been implicated in AMD pathogenesis by damaging RPE cells^[20]^. We found that IL-6 secretion was elevated under our stress conditions, which consisted of combined TBHP and LPS treatment (***Fig. 1C***). However, NMN treatment did not modulate this response.

While our institute has recently focused on identifying functional and nutraceutical natural products using our antioxidant screening assays (*e.g.*, DPPH and ABTS), amla extract was selected as a potent antioxidant (***Fig. 1D***). Although existing literature suggests that amla extract may have anti-inflammatory properties^[10]^, evidence regarding its role in ocular health remains limited. Therefore, we examined whether amla extract could mitigate the stress induced by TBHP and LPS in RPE cells. When co-treated with NMN under these conditions, amla extract markedly reduced IL-6 levels in a dose-dependent manner (***Fig. 1C***).

Amla extract is known to contain various bioactive compounds^[10]^, including quercetin and ascorbate. In a preliminary experiment, we also found that amla extract contains abundant vitamin C (***Supplementary Fig. 1***). While ascorbate markedly reduced IL-6 secretion from ARPE-19 cells, quercetin did not significantly suppress this pathological condition (***Fig. 1E***). Based on this finding, we propose that vitamin C in amla extract may exert anti-inflammatory effects in ARPE-19 cells by suppressing IL-6 secretion.

The RPE is a monolayer of cells in the eye that plays crucial roles in maintaining retinal structure and function. Specifically, RPE cells closely interact with the outer retina (containing photoreceptors) by supplying necessary nutrients, regulating ion homeostasis, and clearing metabolic waste and excess byproducts^[21]^. Consequently, RPE dysfunction and/or degeneration are considered initiating or early events in the pathogenesis of various retinal diseases, including AMD.

Oxidative stress and inflammation are known to induce RPE degeneration^[20]^. Therefore, we applied TBHP (a general oxidative stress inducer) and LPS (a well-known pro-inflammatory factor) together to induce complex damage in RPE cells. This combined treatment disrupted NAD^+^ metabolism by reducing intracellular NAD^+^ levels. Given that NAD^+^ depletion has been detected in aging and metabolic diseases^[3]^, NMN supplementation to restore NAD^+^ levels is meaningful for suppressing RPE dysfunction induced by oxidative stress and inflammation.

Inflammation of RPE cells is another key factor in the context of AMD development and progression. RPE cells can produce various pro-inflammatory cytokines, such as IL-1α, IL-1β, IL-2, IL-5, IL-6, IL-8, and IL-12^[22]^. In particular, senescent RPE cells upregulate the inflammatory cytokine IL-6^[23]^. We found that the combined stress induced by TBHP and LPS increased IL-6 secretion from ARPE-19 cells. Although NMN showed no direct effect, amla extract and its component (vitamin C) reduced IL-6 secretion. Supplementation with vitamin C has been widely reported to reduce IL-6 levels^[24]^. Therefore, co-administration of NMN and vitamin C may exert both antioxidant and anti-inflammatory effects in RPE cells.

As a pilot study, we have provided preliminary evidence for the therapeutic effects of NMN and amla (with vitamin C) on mitigating oxidative stress- and inflammation-induced injury in human RPE cells. Various preventive and protective aspects, such as the secretion of other pro-inflammatory cytokines, cellular ROS levels, or caspase activities, can be evaluated using our combined strategy of NMN and vitamin C in RPE cells both *in vitro* and *in vivo*. Moreover, the specific roles of sirtuins together with vitamin C in regulating oxidative stress and inflammation warrant further exploration in RPE cells. These aspects represent key directions for future research and are acknowledged as limitations of our current study. In conclusion, the co-administration of NMN and amla (particularly vitamin C) holds promise as a therapeutic approach for alleviating RPE dysfunction and damage, and merits further investigation as a potential strategy for the treatment of AMD.

The current study was supported by the Basic Science Research Program through the National Research Foundation of Korea (NRF), funded by the Ministry of Education (Grant No. RS-2021-NR060133 to S.S.L.).

Yours sincerely,

Deokho Lee^1^, Jolly Shamsun Nahar^2^, Sichan Kim^2^, Soon Sung Lim^1,2,^^✉^

^1^The Korean Institute of Nutrition, Hallym University,

Chuncheon, Gangwon-do 24252 

Republic of Korea.

^2^Department of Food Science and Nutrition, Hallym University,

Chuncheon, Gangwon-do 24252 

Republic of Korea.

^✉^Corresponding author: Soon Sung Lim, E-mail: limss@hallym.ac.kr, ORCID: 0000-0003-4548-1285.

## Additional information

The online version contains supplementary materials available at http://www.jbr-pub.org.cn/article/doi/10.7555/JBR.39.20250452?pageType=en.
