# Supplementary material for: Therapeutic effects of nicotinamide mononucleotide and Indian gooseberry in oxidative stress and inflammation-induced damage on human retinal pigment epithelial cells: A pilot study
Source: J Biomed Res. 2026 May 21;40(3):327–31. doi: 10.7555/JBR.39.20250452 (PMC13231348; doi:10.7555/JBR.39.20250452)
Supplement: Supplementary file 1 — The online version contains supplementary materials available at http://www.jbr-pub.org.cn/article/doi/10.7555/JBR.39.20250452?pageType=en. [file jbr-40-3-327-S1.pdf]

Available online at www.jbr-pub.org.cn

Open Access at PubMed Central

JBR

Journal of Biomedical Research, 2026 40(3): 1–1

Supplementary Data

Therapeutic effects of nicotinamide mononucleotide and Indian gooseberry in oxidative stress and inflammation-induced damage on human retinal pigment epithelial cells: A pilot study

Deokho Lee[1], Jolly Shamsun Nahar[2], Sichan Kim[2], Soon Sung Lim[1,2,✉]

[1]The Korean Institute of Nutrition, Hallym University, Chuncheon, Gangwon-do 24252, Republic of Korea; [2]Department of Food Science and Nutrition, Hallym University, Chuncheon, Gangwon-do 24252, Republic of Korea.

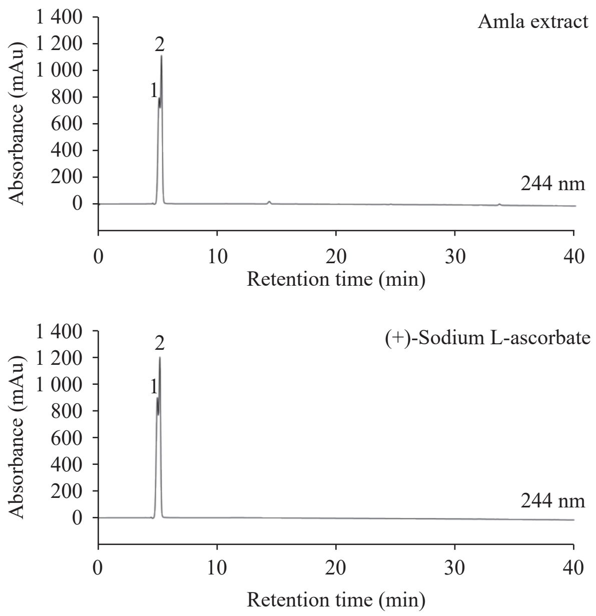

Supplementary Fig. 1 **Identification and quantitative analysis of (+)-sodium L-ascorbate in amla extract.** High-performance liquid chromatography (HPLC) chromatograms of amla extract and (+)-sodium L-ascorbate standard (500 µg/mL). The analysis wavelength was set at 244 nm, corresponding to the maximum absorption (λ_{max}) determined by UV-Vis spectral analysis, ensuring optimal sensitivity for quantification. Two major peaks in amla extract, observed at retention times of 5.029 min (Peak 1) and 5.233 min (Peak 2), were identified as (+)-sodium L-ascorbate by confirming consistent retention times and identical UV-Vis absorption spectral profiles with the reference standard. A calibration curve was established using (+)-sodium L-ascorbate standards prepared *via* serial dilution (50, 100, and 500 µg/mL), yielding a linear regression equation of $y = 45.273x + 1\,039.8$ ($R^2 = 0.996\,9$). Quantitative assessment revealed a high (+)-sodium L-ascorbate content of approximately 440.48 mg/g (44.05% w/w) in our amla extract. Abbreviation: mAu, milli-absorbance units.

✉Corresponding author: Soon Sung Lim, Hallym University, 1 Hallymdeahak-Gil, Chuncheon, Gangwon-do 24252, Republic of Korea. E-mail: limss@hallym.ac.kr. ORCID: 0000-0003-4548-1285.

Received: 21 October 2025; Revised: 16 January 2026; Accepted: 21 January 2026; Available online: 28 January 2026; Published date: 21 May 2026

CLC number: R774.1, Document code: B

The authors reported no conflict of interests.

This is an open access article under the Creative Commons Attribution (CC BY 4.0) license, which permits others to distribute, remix, adapt and build upon this work, for commercial use, provided the original work is properly cited.

© 2026 by Journal of Biomedical Research.

https://doi.org/10.7555/JBR.39.20250452
